# Supplementary material for: Comparison of different regimens with or without fluoroquinolone in isoniazid-resistant tuberculosis: A multicenter cohort study
Source: PLoS One. 2022 Aug 18;17(8):e0273263. doi: 10.1371/journal.pone.0273263 (PMC9387806; doi:10.1371/journal.pone.0273263)
Supplement: Attachment — Submitted filename: resubmission_response_220802+R2.docx [file pone.0273263.s003.docx]

**Reviewer #2:** I thank the authors for the numerous edits to the manuscript, which I think is much improved. I have 1 request, based on the response to the comments on regimen duration. The 6REZ regimen was considered extended if >10 months and the 2/7 regimen considered extended if >13 months. I would rename these regimens: 6-9REZ and 2REZ/7-10RE -- just as we do with standardized short MDRTB regimens.

*[response]* I truly appreciate another opportunity to revise the manuscript. I understood the valuable comment and renamed the regimens in the manuscript and figures, accordingly. I changed 6REZ to 6-9REZ. I changed 2REZ/7RE to 2REZ/7-10RE.
