# Supplementary material for: Comparison of different regimens with or without fluoroquinolone in isoniazid-resistant tuberculosis: A multicenter cohort study
Source: PLoS One. 2022 Aug 18;17(8):e0273263. doi: 10.1371/journal.pone.0273263 (PMC9387806; doi:10.1371/journal.pone.0273263)
Supplement: Attachment — Submitted filename: resubmission_response_220804+R3.docx [file pone.0273263.s004.docx]

Additional Editor Comments:

Regarding treatment outcome (lines 104-110, page 5) in materials and methods, the abstract (lines 35-38, page 2) should be corrected accordingly. Therefore, “Baseline regimens were classified into two groups, namely 6-month rifampicin, ethambutol, and pyrazinamide (6-9REZ) and a combination regimen of 2-month rifampicin, ethambutol, pyrazinamide and 7-month rifampicin and ethambutol (2REZ/7-10RE).” in the abstract should be corrected to “Baseline regimens were classified into two groups, namely 6-9 month rifampicin, ethambutol, and pyrazinamide (6-9REZ) and a combination regimen of 2-month rifampicin, ethambutol, pyrazinamide and 7-10 month rifampicin and ethambutol (2REZ/7-10RE).”.

[response] Thank you for your comment. We changed it as follows.

“Baseline regimens were classified into two groups, namely 6–9-month rifampicin, ethambutol, and pyrazinamide (6-9REZ) and a combination regimen of 2-month rifampicin, ethambutol, pyrazinamide and 7–10-month rifampicin and ethambutol (2REZ/7-10RE).”
